# Quadruple Assessment of Colorectal Anastomosis after Laparoscopic Rectal Resection: A Retrospective Analysis of a Propensity-Matched Cohort

**DOI:** 10.3390/jcm13175092

**Published:** 2024-08-27

**Authors:** Filippo Carannante, Guglielmo Niccolò Piozzi, Valentina Miacci, Gianfranco Bianco, Gennaro Melone, Vincenzo Schiavone, Gianluca Costa, Marco Caricato, Jim S. Khan, Gabriella Teresa Capolupo

**Affiliations:** 1 UOC Chirurgia Colorettale, Fondazione Policlinico Universitario Campus Bio-Medico, Via Àlvaro del Portillo 200, 00128 Rome, Italy; valentina.miacci@unicampus.it (V.M.); gianfranco.bianco@unicampus.it (G.B.); gennaro.melone@unicampus.it (G.M.); g.costa@policlinicocampus.it (G.C.); m.caricato@policlinicocampus.it (M.C.); g.capolupo@policlinicocampus.it (G.T.C.); 2 Research Unit of Surgery, Department of Medicine and Surgery, Università Campus Bio-Medico, Via Àlvaro del Portillo 200, 00128 Rome, Italy; 3 Department of Colorectal Surgery, Portsmouth Hospitals University NHS Trust, Portsmouth PO6 3FT, UK; guglielmopiozzi@gmail.com (G.N.P.); jim.khan@porthosp.nhs.uk (J.S.K.); 4 Advanced Biomedical Sciences Department, “Federico II” University, AOU “Federico II”, Via S. Pansini 5, 80131 Naples, Italy; vincenzo.schiavone@policlinicocampus.it; 5 Department of Life Science, Health and Health Professions, Link Campus University, 00165 Rome, Italy; 6 Faculty of Science and Health, University of Portsmouth, Portsmouth PO1 2UP, UK

**Keywords:** anastomotic leak, colorectal anastomosis, indocyanine green, rectal cancer, quadruple assessment

## Abstract

**Background:** Anastomotic leakage (AL) is one of the most feared complications in colorectal surgery, with an incidence of 12–39% and associated risk of mortality of 2–24%. The causes of AL and the ways to prevent it are currently under investigation. This study aims to verify if a quadruple assessment of colorectal anastomosis could reduce AL incidence. **Methods:** A retrospective analysis of prospectively collected data on rectal cancer surgery performed from January 2015 to December 2017 and from January 2021 to December 2023 at a tertiary referral cancer centre was performed. Demographics, clinicopathological features, short-term outcomes, recurrences, and survival were investigated. **Results:** A total of 293 patients were enrolled. AL incidence was lower in the quadruple assessment group than in the control group, reaching a statistically significant result (7.7% vs. 16%; *p* = 0.001). This result was also confirmed after a propensity score match analysis (PSM), in which the AL rate was lower in the quadruple assessment group (5.4% vs. 12.3%; *p* = 0.01). **Conclusions:** This study shows how the systematic implementation of a quadruple assessment when performing a colorectal anastomosis could increase awareness on anastomotic success and reduce the incidence of AL.

## 1. Introduction

Anastomotic leak (AL) is one of the most feared complications in colorectal surgery, with a reported incidence of 12–39% and an associated risk of mortality of 2–24% [1]. AL is associated with increased morbidity, higher risk of cancer recurrence [2], reduced long-term quality of life [3], and increased incidence of a permanent stoma [2,4]. The risk of AL is associated with the height of the rectal dissection, increasing from low anterior resection to ultralow anterior resection, and to ileoanal and coloanal anastomosis.

Anastomosis between two intestinal layers has been performed since 1823 [5], with the first reported death due to an AL in 1899 [5]. Since then, there has been a debate over the definition of AL, which is still controversial. Due to the use of different definitions of AL, considerable variations in AL rates are reported in the literature [6]. Generally, in accordance with the United Kingdom Surgical Infection Study Group, AL includes all conditions characterised by clinical or radiologic features of anastomotic dehiscence [7]. To facilitate comparing the different studies on AL, specific guidelines on AL definitions were published by the International Study Group of Rectal Cancer Criteria. According to these guidelines, AL was divided into the following: Grade A, detected by radiographic findings of perianastomotic fluid collection or leakage of contrast medium through the anastomosis without the patient showing clinical signs (grade A is not in need of active therapeutic intervention); Grade B, AL is in need of therapeutic interventions such as antibiotics and/or percutaneous drainage; Grade C, AL requires surgery [8,9].

Several patient-related factors, perioperative factors, and technical considerations have been recognised as risk factors for AL. Different methods have been proposed to prevent AL [10,11,12,13], but the choice of method is dependent on multiple factors, which are divided in modifiable and fixed categories. Improving our knowledge about modifiable risk factors could help in reducing AL incidence. In this study, we have assessed the use of a quadruple control composed of an air leak test, indocyanine green fluorescence angiography (ICGFA) used to decide the proximal resection margin and to evaluate the rectal stump; endoscopic visualization with ICGFA; and the inspection of both tissue rings (‘doughnuts’) after the creation of a circular stapled anastomosis. 

This study aims to verify the role of a quadruple control assessment of colorectal anastomosis in reducing the incidence of AL and the overall short-term post-operative complications after rectal cancer resection.

## 2. Materials and Methods

A retrospective analysis was performed on all consecutive patients undergoing oncological restorative anterior rectal resection from January 2015 to December 2017 and from January 2021 to December 2023 at a tertiary referral Centre.

Patients who had been diagnosed with primary rectal tumours (histologically found) located within 15 cm from the anal verge, and who had undergone surgery with an open or minimally invasive approach, were included in this study. 

The data were extracted from a prospectively maintained colorectal cancer database. 

The inclusion criteria were as follows: (1) rectal resection; (2) elective surgery; (3) histological evidence of adenocarcinoma; and (4) no previous history of colorectal cancer. The exclusion criteria were as follows: (1) emergency surgery; (2) transanal minimally invasive surgery (TAMIS) or transanal excision (TAE), i.e., surgical interventions that consist of local mass excision with a transanal approach; (3) hereditary colorectal cancer; (4) a history of inflammatory bowel disease; (5) combined resection of other major organs (i.e., lungs and liver); (6) a history of bone marrow-related disease; and (7) a history of chronic renal failure. 

Pelvic magnetic resonance imaging (MRI) and chest and abdominal computed tomography (CT) were used to stage the disease in all patients. Patients with locally advanced mid–low rectal tumours (T3-4 and/or N+) underwent neoadjuvant chemoradiotherapy (nCRT) followed by total mesorectal excision (TME). 

From January 2020, a quadruple assessment technique was performed for colorectal anastomosis control in our surgical practice. The technique included the following steps: (1) Indocyanine green fluorescence angiography (ICGFA) used to decide the proximal resection margin and to evaluate the rectal stump. Stapling was performed after confirmation of adequate perfusion. The adequate perfusion of the final colorectal anastomosis, before removing the trocars, was also checked through ICGFA. Any defect was addressed by reconstruction of the anastomosis. (2) Visual inspection of the anastomotic rings (“doughnuts”). (3) An air leak test: the pelvis was filled with saline, and the bowel proximal to the anastomosis gently occluded, and the patient was placed in a neutral or reverse Trendelenburg position. A urinary catheter was inserted through the anus and air was insufflated to carry out the leak test. Any defect was addressed by reinforcement or reconstruction of the anastomosis. (4) Finally, by using an ICGFA scope, an endoscopic evaluation of the colorectal anastomosis was performed to check for mucosa perfusion and early bleeding.

Postoperative complications were analysed to compare the short-term postoperative outcomes between the two groups (pre- and post-quadruple control). Surgical site infection, urinary tract infection, pneumonia, postoperative bleeding, anastomotic leakage, reoperation, readmission within 30 days after surgery, and 30-day mortality were compared, respectively. The Clavien–Dindo classification [14] was used to evaluate the severity of the complications.

Anastomotic leak was detected and classified as stated by the International Study Group of Rectal Cancer criteria [8]. Grade A anastomotic leaks are detected by radiographic findings of perianastomotic fluid collection or leakage of contrast medium through the anastomosis without the patient showing clinical signs (grade A is not in need of active therapeutic intervention). Grade B leakage requires therapeutic interventions such as antibiotics and percutaneous drainage. Grade C anastomotic leakage requires surgery. When postoperative clinical symptoms (fever, abdominal pain, ileus) and/or abnormal laboratory tests (leukocytosis, C-reactive protein) were detected, the patient underwent a CT scan assessment in order to identify AL. All anastomotic dehiscence with leakage into the pelvic cavity and isolated pelvic abscesses with no evidence of fistula were considered ALs. This study was approved by our institutional editorial board.

## 3. Statistical Analysis

Patients’ characteristics were summarised using basic descriptive statistics. Continuous variables were presented as mean ± standard deviation values and compared using a *t*-test on individual samples. For categorical data, the χ^2^ test was used, and the results are expressed as percentages. All statistical analyses were performed using the SPSS Statistics for Windows version 24.0 software (IBM Corp., Armonk, NY, USA). Nearest neighbour propensity score matching (PSM) extracted 1:1 matched pairs of subjects from the quadruple control group or the non-quadruple control group concerning the patient-, tumour-, and surgery-related characteristics listed in Table 1. Continuous variables are represented by medians (minimum–maximum) or means ± standard deviations (SDs). To analyse the differences in the categorical variables, the chi-squared or Fisher’s exact test was applied. The Wilcoxon rank-sum test was used to compare continuous variables between groups. *p* < 0.05 indicated that the differences between the two groups were statistically significant.

## 4. Results

A total of 293 patients who underwent restorative rectal cancer surgery were included. The patients’ baseline characteristics and perioperative features are reported in Table 1. Tumours were detected in the mid and low rectum in 52.9% of patients. AL occurred in 35 patients (14.6%). Globally, 49.5% of patients received neoadjuvant radiation. The mean operative time was 270 ± 56 min. The intraoperative plan was changed after quadruple control in 11 (7.7%) patients (Table 1).

AL occurred less in the quadruple assessment group than in the control group, reaching a statistically significant result (7.7% vs. 16%; *p* = 0.001).

Due to group heterogeneity, a PSM was performed (Table 2). A 1:1 PSM cohort including 130 patients was created for each group. Patients were equally distributed between the groups for mean age, BMI, ASA score, tumour location, and comorbidity rate. Similarly, no differences in nCRT and surgical parameters were recorded.

AL incidence after PSM occurred less in the quadruple control group than in the control group, reaching a statistically significant correlation (5.4% vs. 12.3%; *p* < 0.001). Length of stay was lower in the quadruple control group, but not statistically significant, while the overall short-term outcomes were similar for both groups.

## 5. Discussion

AL is one of the most dreaded complications in colorectal surgery. In 2017, Khan et al. described their triple control assessment of colorectal anastomosis after robotic anterior resection of the rectum [15]. Two years later, Wexner et al. [16] published their standard technique of quadruple assessment of colorectal and coloanal anastomosis: an air leak test, endoscopic visualisation, an assessment of perfusion with ICGFA, and an inspection of both tissue rings. This control has been adopted routinely in our institution since 2020 [17].

The use of ICGFA is spreading globally. In the last few years, several studies have reported the role of ICGFA to evaluate the perfusion of both bowel stumps used to create an anastomosis [18,19,20,21]. Ischemia of the colorectal anastomosis has been recognised to play a fundamental role in the development of AL, even if the anastomosis appears anatomically appropriate when the surgeon performs it. Assessment of the perfusion with ICGFA has proven to be effective in changing intra-operative decisions, going as far as creating a new anastomosis altogether, if the perfusion is not optimal [22].

Adequate perfusion is a key factor to consider when evaluating the quality of the anastomosis, but there are many causes for AL; however, when a leak does occur, it is most likely in a multifactorial setting. In this case, the safety and feasibility of ICGFA to detect bowel perfusion during surgery was confirmed in several studies [8,12,13,23,24]. However, the real clinical benefits of ICGFA and its impact on AL are still debated. Emile et al. [25] performed a systematic review of 27 studies including 8786 patients, reporting changes to the surgical plan regarding the level of transection and anastomosis based on the findings made through ICG in 331/3614 patients (9.1%). The rates of patients in which the level of transection was changed ranged from 0.6% to 28.7% across studies, and all changes were associated with the proximal transection.

A recent analysis by Arpaia et al. [26] established a system based on machine learning classifiers able to help surgeons in the operating theatre. This support, based on a decision-making system, is able to automatically evaluate if the quality of the perfusion is estimated to be enough after the injection of ICG. ICG is currently evaluated only qualitatively and subjectively by the surgeon, based on experience, and there are no systems or techniques used to currently quantify it. It is interesting to investigate the application of ICG and artificial intelligence in order to objectively detect bowel perfusion.

To date, four RCTs have been performed. De Nardi et al. [27] had a limited number of participants and, consequently, insufficient power, recruiting 240 patients. Furthermore, in addition to rectal cancer, this RCT included patients with left-sided colonic cancer. In the PILLAR III [28] trial, designed as a phase III trial for low anterior resection with a sample size of 800 patients, enrolment was interrupted at 347 cases and, thus, like the previous study, this study was also underpowered. The FLAG trial [29] established that ICG remarkably reduced AL. However, the FLAG randomised trial enrolled 377 elective patients with either malignant or benign sigmoid or rectal cancer, and targeted only rectal cancer located higher than 12 cm from the anal verge. The EssentiAL trial [30] enrolled 850 patients and included only patients with rectal cancer localised 12 cm or less from the anal verge. This study was the first phase III RCT conducted to demonstrate the superiority of a blood flow assessment using ICGFA compared with a standard blood flow assessment in minimally invasive surgery for rectal cancer. The authors hypothesised a reduction in the AL incidence rate by 6%, as stated by previous meta-analyses [10]. Notwithstanding, they achieved a reduction rate of 4.2%, which was less than the expected reduction rate. Although the use of ICG significantly reduced the rate of AL, the target hypothetical reduction of 6% was not reached.

The IntAct trial is a prospective, unblinded, multicentre randomised controlled trial that will observe AL rates at 90 days post-operatively among 880 patients undergoing minimally invasive low anterior resection for rectal cancer with or without intraoperative ICGFA. The patient recruitment ended in August 2023, and the results are awaited.

Another point of discussion should be on our use of an ileostomy after the construction of an anastomosis. In our centre, 56.3% of all patients received a protective ileostomy, and of those, 5.4% experienced an AL. We performed ileostomy in all patients with a high risk of AL, so all patients with TME resection. A study by Garg et al. [31] showed how a protective ileostomy lowered the risk of AL by one-third in 390 patients who had undergone a protective diversion ileostomy at the time of surgery (low anterior resection) and 378 who had not, resulting in a total of 768 patients, all of whom were included in the meta-analysis. The fashioning of an ileostomy significantly decreased AL rates (*p* < 0.000) and reoperation rates (*p* < 0.000). Performing an ileostomy reduces the complication rate if AL happens, and this could be undetected (non- or poorly symptomatic AL). Although controversy remains regarding whether it may affect complications after surgery, something we think is worth mentioning is nCRT. This may create local rectal tissue injury and influence anastomosis healing. Yang et al. [32] show that undergoing nCRT causes a statistically significant increase in the incidence of postoperative complications following a colorectal surgery such as AL, as well as an increased risk of pelvic abscess and wound infection. We always consider nCRT as a complicating factor prior to surgery due to possible adhesions, tissue injury, and reduced angiogenesis caused by radiotherapy, which can have indirect consequences.

AL may be firmly influenced by physical factors, such as ultra-low anastomosis and delayed wound healing after irradiation, causing inadequate blood flow. According to the PILLAR III trial [13], 64.6% of patients underwent nCRT, and 83.0% with lower/mid rectal cancer required low anastomosis, and in the EssentiAL trial [8], 83.5% of patients underwent nCRT and all patients had lower/mid rectal cancer requiring a low anastomosis.

These studies had considerable limitations; for example, there was a risk of observational bias due to the knowledge of the study group assignments in both patients and surgeons, the results were limited to the selected population, the anastomosis method was not the same in all study patients, and there was an absence of provisions concerning left colic artery preservation or diverting stoma.

Regarding the inspection of the doughnuts, the first report was by Goriainov et al. [33], who inspected the doughnuts before evaluating the anastomosis with an air leak test. However, the only evaluation of circumferentially full-thickness tissue doughnuts does not guarantee the absence of postoperative AL [34].

Another important aspect of anastomosis evaluation is the air leak test, the most frequent intraoperative test performed to mechanically detect inadequate colorectal anastomosis that needs intraoperative repair. A recent meta-analysis [35] concludes that the use of an air leak test did not significantly reduce the incidence of AL, but confirms prior findings that a positive air leak test might be associated with a higher risk of AL [36]. Other studies [37,38] reported the possibility of performing a reverse air leak test for very low coloanal anastomosis at the dentate line or when performing a transanal anastomosis, where the standard air leak test cannot be performed.

It is debated whether the intraoperative evaluation of anastomosis is useful, while the use of ICGFA is becoming increasingly widespread.

Finally, the impact of AL on long-term oncological outcomes is another important topic. Several studies have suggested [39,40] that AL could be associated with rectal cancer’s local recurrence (LR). The mechanism by which AL increases LR after rectal cancer surgery remains unclear. Postoperative sepsis may cause an inflammatory response. The literature suggests that the systemic inflammatory response is involved in the progression of metastatic disease in patients with colorectal cancer [41]. In addition to this, it has been reported that postoperative sepsis could lead to a period of immunosuppression following proliferation of metastatic tumour cells [42]. On the other hand, AL might lead to the local implantation of viable cancer cells at the anastomotic site at the time of surgery [43]. Finally, survival in patients with colorectal cancer can be influenced by a delayed adjuvant treatment due to a prolonged length of hospital stay [44].

The present study has several limitations. This is a retrospective and monocentric analysis of a relatively small number of patients. Prospective, randomised studies are important to evaluate, with stronger evidence, the present results.

The systematic use of all four assessment techniques for intraoperative evaluation could minimise both the incidence of AL and the frequency of the derivative stoma. All these procedures, considered individually, may have little impact on the incidence of AL, but, together, this assessment could help to improve and, hopefully, reduce one of the “Achilles heels” of colorectal surgery.

## 6. Conclusions

The systematic use of a quadruple control assessment when performing a colorectal anastomosis could reduce the incidence of AL and help surgeons discover complications intraoperatively. Every step of this assessment is very important to identify possible issues with the integrity of the bowel anastomosis and could allow surgeons to resolve anastomotic defects early before the insurgence of clinical complications.

## Figures and Tables

**Table 1:** Baseline characteristics of the study population. ASA: American Society of Anesthesiologists; BMI: Body Mass Index; nCRT: neoadjuvant chemoradiotherapy.

	Total *n* = 293	Quadruple Control Group *n* = 143	Control Group *n* = 150	*p*
Mean age (±SD)	68.5 (±11.5)	65.17 (±13.25)	68.50 (±10.25)	0.84
Gender, %				0.07
Male	113 (38.5)	51 (35.6)	62 (43.4)	
Female	180 (61.5)	92 (64.4)	88 (56.6)	
ASA score, %				0.49
1/2	157 (54.2)	75 (52.4)	82 (55.1)	
3/4	136 (45.8)	68 (47.6)	68 (44.9)	
BMI (Kg/m^2^), mean (±SD)	25.4 (±4.08)	25.81 (±4.19)	25.16 (±2.99)	0.52
Comorbidity, %	201 (68.6)	98 (68.5)	103 (68.6)	0.58
Tumour distance from AV				0.56
>10 cm	138 (47.1)	63 (45.7)	75 (49.5)	
5.1–10 cm	92 (31.4)	49 (34.4)	43 (29)	
<5 cm	63 (21.5)	31 (21.5)	32 (21.2)	
nCRT, %	145 (49.5)	71 (49.6)	74 (49.3)	0.88
Surgical approach, %				0.81
Open	97 (33.2)	49 (34.4)	48 (33)	
Laparoscopic	192 (65.6)	92 (64)	100 (66)	
Robotic	4 (1.2)	2 (1.6)	2 (1)	
Diverting ileostomy, %	165 (56.3)	85 (59.4)	80 (53.3)	0.43
Time of surgery (±SD)	270 ± 56	273 ± 87	265 ± 94	0.54
Anastomotic leakage, %	35 (14.6)	11 (7.7)	24 (16)	0.001
Change in intraoperative plan after ICGFA, %	7 (4.9)	7 (4.9)	0 (0)	-
Change in intraoperative plan after doughnuts inspection, %	0 (0)	0 (0)	0 (0)	-
Change in intraoperative plan after Air leak test, %	3 (2.1)	3 (2.1)	0 (0)	-
Change in intraoperative plan after endoscopic evaluation, %	1 (0.7)	1 (0.7)	0 (0)	-
Change in intraoperative plan after quadruple control, %	11 (7.7)	11 (7.7)	0 (0)	-
Length of stay, days (±SD)	9.3 ± 6.4	8.2 ± 5.7	10.2 ± 7.1	0.09
30-day mortality, %	9 (3)	5 (3.5)	4 (2.6)	0.65

**Table 2:** Baseline characteristics of the study population after propensity score matching.

	Quadruple Control Group *n* = 130	Control Group *n* = 130	*p*
Mean age (± SD)	70.79 ± 10.95	68.12 ± 10.52	0.184
Gender, %			0.078
Male	71 (54.79)	85 (65.75)	
Female	59 (45.21)	45 (34.25)	
ASA score, %			0.49
1/2	75 (57.7)	72 (55.4)	
3/4	55 (42.3)	58 (44.6)	
Comorbidity, %	85 (65.4)	88 (67.7)	0.58
Tumour distance from AV, %			0.56
>10 cm	44 (34.25)	41 (31.51)	
5.1–10 cm	50 (38.36)	57 (43.84)	
<5 cm	36 (27.4)	32 (24.7)	
nCRT, %	60 (46.1)	63 (48.5)	0.88
Diverting ileostomy, %	62 (47.7)	65 (50)	0.43
Time of surgery (±SD)	253 ± 57	265 ± 64	0.44
Anastomotic leakage, %	7 (5.4)	16 (12.3)	0.001
- >10 cm	0	2	
- 5–10 cm	2	5	
- <5 cm	5	9	
- With ileostomy	7	14	
Bleeding, %	1 (0.08)	2 (1.5)	0.56
Ileus, %	4 (3.1)	3 (2.3)	0.6
Surgical site infection, %	5 (3.8)	3 (2.3)	0.23
Pneumonia, %	3 (2.3)	4 (3.1)	0.6
Urinary tract infection, %	2 (1.5)	3 (2.3)	0.32
Lenght of stay, days (±SD)	8.2 ± 5.7	10.2 ± 7.1	0.09
30-day mortality	2 (1.5)	2 (1.5)	0.65

## Data Availability

The data presented in this study are available on request from the corresponding author.
